# Streamlining Acute Abdominal Aortic Dissection Management—An AI-based CT Imaging Workflow

**DOI:** 10.1007/s10278-024-01164-0

**Published:** 2024-06-12

**Authors:** Anish Raj, Ahmad Allababidi, Hany Kayed, Andreas L. H. Gerken, Julia Müller, Stefan O. Schoenberg, Frank G. Zöllner, Johann S. Rink

**Affiliations:** 1 Computer Assisted Clinical Medicine, Medical Faculty Mannheim, Heidelberg University, Theodor-Kutzer-Ufer 1-3, D-68167 Mannheim, Germany; 2 Mannheim Institute for Intelligent Systems in Medicine, Medical Faculty Mannheim, Heidelberg University, Theodor-Kutzer-Ufer 1-3, D-68167 Mannheim, Germany; 3 Department of Radiology and Nuclear Medicine, University Medical Center Mannheim, Theodor-Kutzer-Ufer 1-3, D-68167 Mannheim, Germany; 4 Department of Surgery, Medical Faculty Mannheim, University Medical Center Mannheim, Heidelberg University, Theodor-Kutzer-Ufer 1-3, D-68167 Mannheim, Germany; 5 Mediri GmbH, Eppelheimer Straße 13, D-69115 Heidelberg, Germany

**Keywords:** Aortic dissection, Convolutional neural network, Deep learning, Computed tomography, Abdomen

## Abstract

Life-threatening acute aortic dissection (AD) demands timely diagnosis for effective intervention. To streamline intrahospital workflows, automated detection of AD in abdominal computed tomography (CT) scans seems useful to assist humans. We aimed at creating a robust convolutional neural network (CNN)-based pipeline capable of real-time screening for signs of abdominal AD in CT. In this retrospective study, abdominal CT data from AD patients presenting with AD and from non-AD patients were collected (*n* 195, AD cases 94, mean age 65.9 years, female ratio 35.8%). A CNN-based algorithm was developed with the goal of enabling a robust, automated, and highly sensitive detection of abdominal AD. Two sets from internal (*n* = 32, AD cases 16) and external sources (*n* = 1189, AD cases 100) were procured for validation. The abdominal region was extracted, followed by the automatic isolation of the aorta region of interest (ROI) and highlighting of the membrane via edge extraction, followed by classification of the aortic ROI as dissected/healthy. A fivefold cross-validation was employed on the internal set, and an ensemble of the 5 trained models was used to predict the internal and external validation set. Evaluation metrics included receiver operating characteristic curve (AUC) and balanced accuracy. The AUC, balanced accuracy, and sensitivity scores of the internal dataset were 0.932 (CI 0.891–0.963), 0.860, and 0.885, respectively. For the internal validation dataset, the AUC, balanced accuracy, and sensitivity scores were 0.887 (CI 0.732–0.988), 0.781, and 0.875, respectively. Furthermore, for the external validation dataset, AUC, balanced accuracy, and sensitivity scores were 0.993 (CI 0.918–0.994), 0.933, and 1.000, respectively. The proposed automated pipeline could assist humans in expediting acute aortic dissection management when integrated into clinical workflows.

**Supplementary Information:**

The online version contains supplementary material available at 10.1007/s10278-024-01164-0.

## Introduction

Acute aortic syndrome (AAS) consists of the life-threatening conditions of aortic dissection (AD), intramural hematoma (IMH), and penetrating atherosclerotic ulcer (PAU) [1]. In acute AD, tearing of the aortic vessel intima leads to uncontrolled blood inflow into the aortic wall. Incidence has been reported to range from 2.6 to 7.2 cases per 100,000 person-years and is associated with a reported in-hospital mortality of 30.1% in women and 21.0% in men, creating a substantial healthcare burden [2].

The event of acute AD can cause severe abdominal or back pain; however, in many cases, symptoms are nonspecific and driven by secondary complications like visceral ischemia, resulting in a relatively high rate of patients not initially being suspected of AD, thus receiving abdominal imaging for other reasons [3]. Modern medical management of acute AD aims at early CT-based diagnosis and prompt therapy stratification. Whereas thoracic AD including the ascending part of the vessel (type A) typically requires immediate intervention, AD located more distally in the aorta (type B) in the absence of complications like rupture, malperfusion of visceral organs, spinal ischemia, or lower limb ischemia [4] may be managed conservatively or with elective intervention. In CT imaging, acute AD can in many cases be distinguished from its chronic form [5], bearing the risk of development of abdominal aortic aneurysm (AAA), representing a chronic risk for rupture.

In many cases, the unspecific clinical symptoms and even radiological misdiagnosis under emergency conditions which were reported at 35% for type A AD and 17% for type B AD in a British setting [6] possibly compromise timely suspicion, diagnosis, and treatment of this rare but time-critical medical condition in a substantial proportion of patients [7].

Multiple factors have been addressed to streamline AD management workflows [8]. Artificial intelligence (AI)-based techniques have recently been described as promising tools, capable of detecting critical findings, prioritizing cases accordingly, and eventually reducing delays [9]. Multiple groups have aimed to create algorithms capable of detecting AD mainly in thoracic CT scans. Yi et al. [10] developed a method using a 2.5D U-Net to extract aorta masks and subsequently used a 3D ResNet34 CNN [11], pre-trained on MedicalNet [12], for feature extraction and final prediction via a Gaussian Naive Bayes algorithm by combining radiomics and CNN features. The results showed high performance on internal and external datasets (AUC = 0.948, sensitivity = 0.862, specificity = 0.923 for internal (341 patients); AUC = 0.969, sensitivity = 0.978, specificity = 0.554 for external (111 patients)). Despite their robust results on the internal set, they attained low specificity on a small external dataset. Hata et al. [13] utilized a 2D Xception architecture [14], pre-trained on ImageNet [15], to classify AD in non-contrast-enhanced CT scans (170 patients). They achieved an AUC of 0.940, sensitivity of 0.918, and specificity of 0.882 by classifying consecutive slices of the aorta. However, their study’s limitation lies in the lack of external validation, raising concerns about the generalizability of their model. Huang et al. [16] proposed a 2-step hierarchical model involving an attention U-Net for initial AD detection (AD case; if 5 or more slices were detected with AD) followed by a ResNext model [17] for Stanford type classification. Their internal results showed excellent performance (AUC = 0.980, recall = 0.960, specificity = 1.000 for AD detection; AUC = 0.950, recall = 0.947, specificity = 0.953 for Stanford types). Despite high internal metrics, their approach was not tested on an external dataset, questioning its robustness. Harris et al. developed a 2D five-layer CNN as a screening algorithm, yielding a sensitivity of 0.878 and a specificity of 0.960 [18] with a training dataset of 778 patients, and demonstrated a reduction of turnover time (395 s) by prioritizing worklist in a teleradiology setting in the USA. Cheng et al. employed a U-Net to first segment the aorta and then analyze its circularity for AD classification. They obtained a sensitivity of 0.900 and specificity of 0.800 on a comparably small Chinese dataset of *n* = 20 patients (10 AD) [19]. Yellapragada et al. described a wider focussed 3D deep learning model for the detection of AAS solely in CTA scans, trained on a dataset of 3500 cases (500 containing AAS, unclear number of AD cases), overall yielding promising results [20]. Guo et al. [21] segmented the aorta ROI manually and extracted 396 radiomic features, including texture features, gray-level co-occurrence matrix, gray-level run-length matrix, gray-level size zone matrix, form factor features, and histograms. They selected the top 20 features using max-relevance min-redundancy and constructed a radiomic signature via LASSO logistic regression, followed by logistic regression for classification. Their results indicated consistent performance on internal (304 patients, AUC = 0.92, recall = 0.941, specificity = 0.753) and external (74 patients, AUC = 0.90, recall = 0.857, specificity = 0.917) datasets. It is noteworthy that their method involves manual segmentation of the aorta, which is time-consuming. Manual segmentation diminishes the benefits of an automated AD detection algorithm, as the time spent could instead be used by a radiologist to identify dissection cases directly. Additionally, the unavailability of a large external dataset in these studies underscores the necessity of developing robust algorithms that can generalize well across diverse (external) datasets. Furthermore, in the medical domain, the availability of large annotated datasets (for training) is particularly challenging due to factors such as patient privacy concerns, the extensive time required for expert annotation, and the variability in imaging protocols across institutions. Hence, producing a reliable model using a small internal dataset that can be effectively validated on a larger external dataset without manual segmentation is vital.

The aim of this analysis was to develop an easy-to-train convolutional neural network (CNN)-based AI pipeline on a small dataset that can be validated on a large external dataset and capable of real-time screening of all intrahospital abdominal CT scans for signs of AD, even at peak times and independent of acquired contrast phase. High robustness, efficient use of computing power, and a high degree of automation are therefore key requirements.

## Materials and Methods

Possible conflicts of interest have been stated elsewhere. This retrospective study was approved by the local institutional review board (2021–635). Written informed consent was not required due to the retrospective nature of the study population enrolled.

### Data Collection

#### Internal Training and Validation Dataset

Patients presenting with acute AD with abdominal extent between 2010/01/01 and 2021/03/01 were identified in the Radiology Information System (RIS) and included in the study, if an abdominal CT exam had been performed, intentionally regardless of contrast phase or other parameters like image quality or presence of metallic interferences. Patients with preexisting AD and scans not covering the entire abdomen were excluded. To include AD-negative cases, studies from patients with matching contrast phases and patient characteristics were collected similarly, of which some have already been used in a previous, different study [22], representing patient data overlap (*n* = 85). Datasets were randomly split into training (80%; 163 patients) and validation (20%; 32 patients) datasets. The validation set is used only for testing, and we call it internal validation dataset. Patient-specific data was removed.

#### External Validation Dataset

Anonymous external non-synthetic CT data containing healthy aortic vessels and AD were created from the publicly available ImageTBAD [23], AVT [24], and Abdomenct-1 k collections [25]. To avoid data overlap, we discarded the 20 KiTS19 patients from the AVT dataset, since these were included in the Abdomenct-1 k set.

**Fig. 1:**
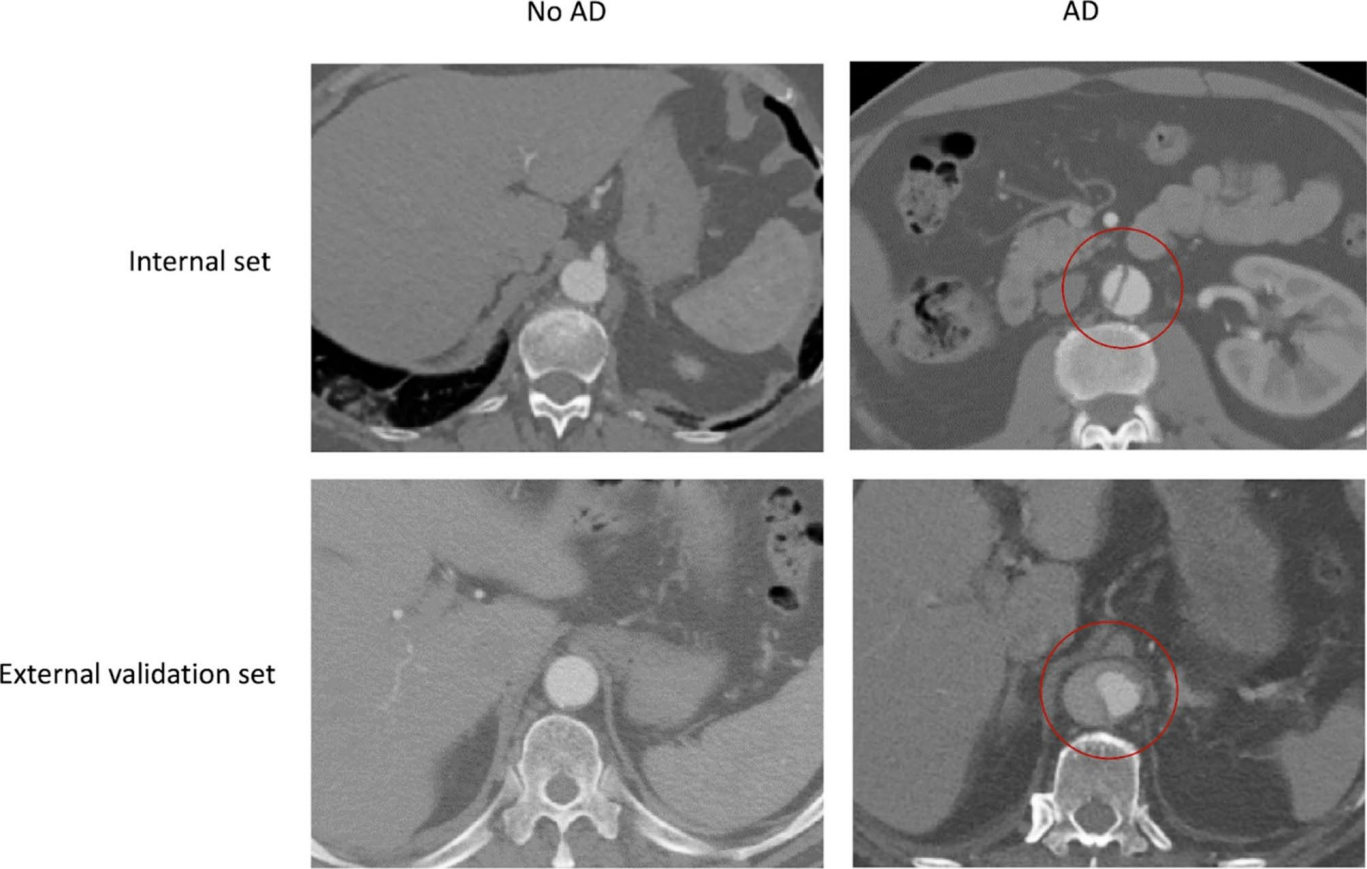
Example images from internal and external datasets. The left column is healthy cases and the right column is aortic dissection (AD) cases

Example images are displayed in Fig. 1.

### Image Annotation Strategy

Annotation was performed in Aycan Osirix (*Aycan Digitalsysteme, Würzburg, Germany*) by an attending radiologist (5 years of experience in cardiovascular imaging) and a separate second reading by a resident (4 years of experience). Cases with disagreements were resolved by a senior physician with > 15 years of experience. Annotation contained age, sex, CT date, contrast phase, presence of metallic interferences, presence of dissection (small and subtle AD were annotated as positive cases), exact dissection extent, occlusion or dissection of side branches (celiac trunk, superior and inferior mesenteric artery, renal arteries, iliac arteries), and presence of signs of visceral ischemia, intramural hematoma, or aortic thrombosis. The external dataset and its annotations were validated.

### Data Preprocessing

**Fig. 2:**
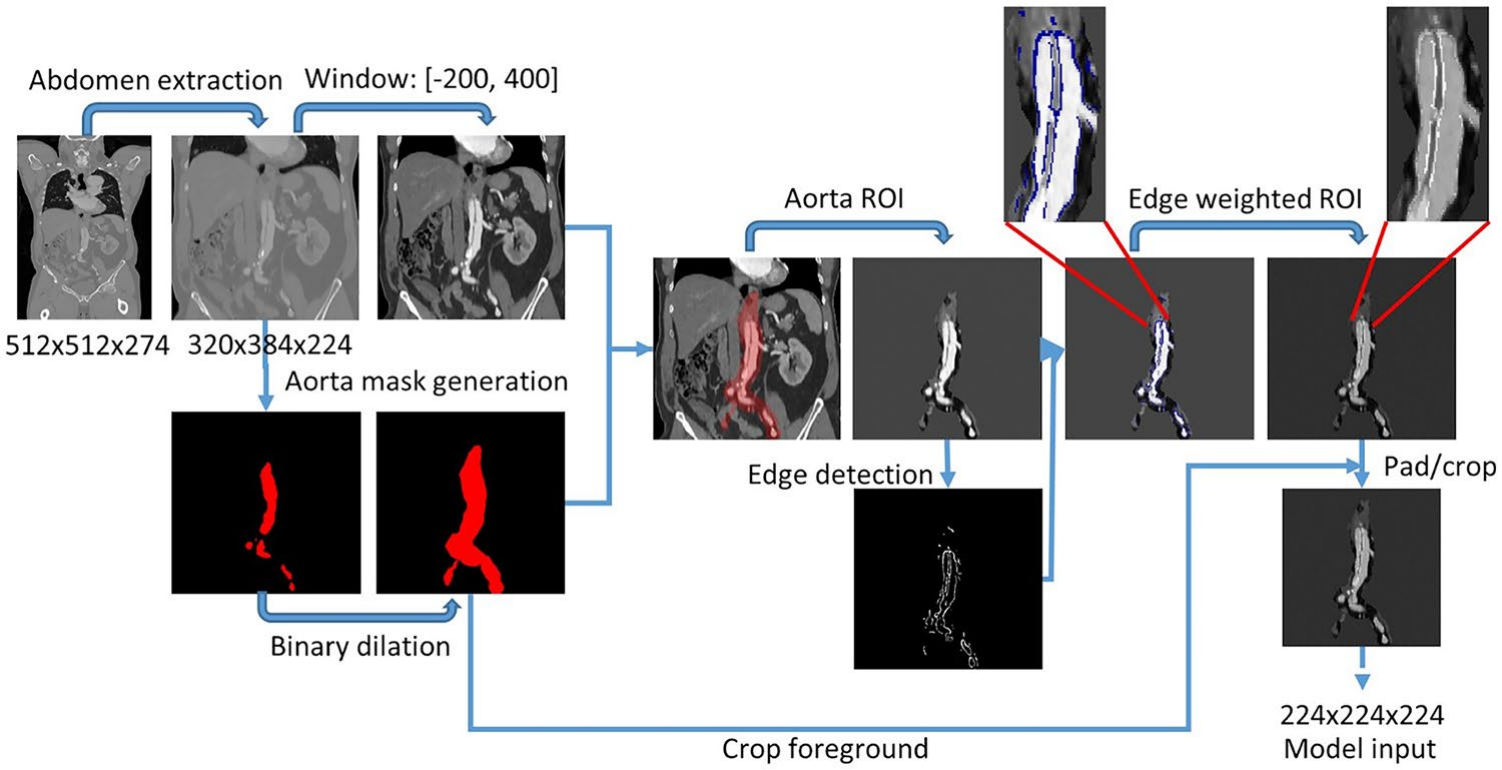
CT volume preprocessing pipeline. The abdominal region is automatically extracted and resized. An aorta mask is generated from this region and then dilated to mask out the aorta region of interest (ROI) from the abdomen region. For highlighting aorta bifurcation in AD cases, edges are extracted using the canny detector. The aorta ROI is weighted down by a factor of 0.6 where there is no edge voxel present. Finally, the edge-weighted aorta ROI is standardized by cropping/padding with the bounding box of the dilated aortic mask

We extract the abdominal region from each CT case using a heuristic implemented in [22]. The algorithm analyzes HU distribution along the z-axis in the soft tissue HU range to establish the upper and lower bounds of the abdomen, determining the abdomen center using high HU values, and extracting a subvolume around it. The extracted subvolumes are resampled to a size of 320 × 384 × 224 voxels with a uniform spacing of 0.9 × 0.9 × 1.5 mm^3^. The intensities are windowed in the [− 200, 400] HU range, corresponding to the soft tissue domain. Next, the CT image is rescaled to be in the range [0, 1]. Subsequently, the mask of the abdominal aorta including iliac arteries is extracted using the TotalSegmentator algorithm [26]. To compensate for the insufficient segmentation quality of dissected vessels, aorta masks were dilated in 10 iterations via a square structuring element with a connectivity of one, ensuring complete coverage. The CT image is then masked using the dilated mask. The dissected aorta has a membrane separating the true and false lumen, which was highlighted by extracting edges (canny detector (σ = 2)) inside the aortic ROI. The subvolume voxels that do not belong to the edges are weighed down with a factor of 0.6, while the edge voxels remain unchanged. We then extract the bounding box of weighted aorta ROI using the dilated mask with a boundary margin of 5 voxels in each direction. Finally, the bounding box is resized with padding or cropping to a standardized size of 224 × 224 × 224 voxels (CNN input) in order to standardize data and minimize the usage of memory. The subvolume of this size is sufficient to encompass the entire aorta ROI (as seen in Fig. 2). The aorta mask is used to create a bounding box, which centers the aorta ROI within this subvolume. The preprocessing pipeline is depicted in Fig. 2. Further data augmentation details are described in the supplemental material.

### Network Architecture

**Fig. 3:**
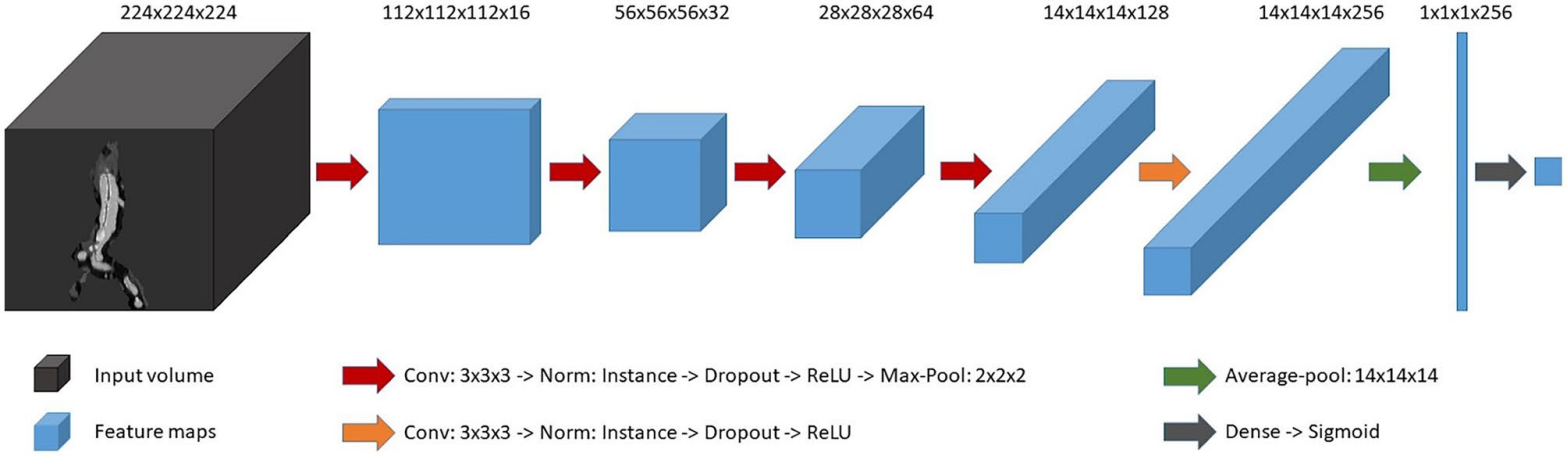
Network architecture for aortic dissection classification. The input volume (edge-weighted aorta ROI) is processed with a CNN to produce a single output in the range [0, 1]. The network consists of convolutional blocks with convolutions of size three, followed by instance norm, dropout, ReLU activation, and max-pool of size two (except in the last convolution block (orange block)). The final feature vector is produced by an average-pooling. It is then processed by a dense layer and a sigmoid activation to produce the output

We construct a CNN comprising 5 convolutional blocks and 1 dense block, depicted in Fig. 3. The network takes as input the masked, edge-weighted aortic ROI. Each convolution block entails a 3 × 3 × 3 convolution, followed by instance normalization, dropout, and ReLU activation. Dropout probabilities for the initial to final convolution blocks are 0.00, 0.05, 0.10, 0.10, and 0.10, correspondingly. Post each of the initial four convolution blocks, a 3D max-pooling operation is performed to downsample the feature maps with a stride of 2. The final feature vector of size 256 is produced using a 3D average-pooling operation with a kernel size of 14. Lastly, the dense block processes this vector, yielding network output through a dense layer followed by sigmoid activation, i.e., a probability score that a patient belongs to either AD or non-AD class. This network was selected after testing various other networks, whose details are provided in the supplements and results.

### Evaluation

To assess the performance of all different networks, the area under the receiver operating characteristic curves (AUCs) were compared to choose the best-performing network for further evaluation. Next, the best network’s performance was further assessed using sensitivity, specificity, balanced accuracy $([Sensitivity + Specificity]/2;$ suited for high class imbalance), and AUC with a 95% confidence interval. We select the binary threshold of our models based on the high sensitivity and balanced specificity on the internal cross validation dataset, i.e., 0.45. Next, we create an ensemble of five models trained on this set to predict the internal validation and external sets based on our set binary threshold. The ensemble combines probabilities (average) for each case in the internal validation and external sets, and we evaluate its performance using the same threshold.

## Results

### Patient and Dataset Characteristics

**Fig. 4:**
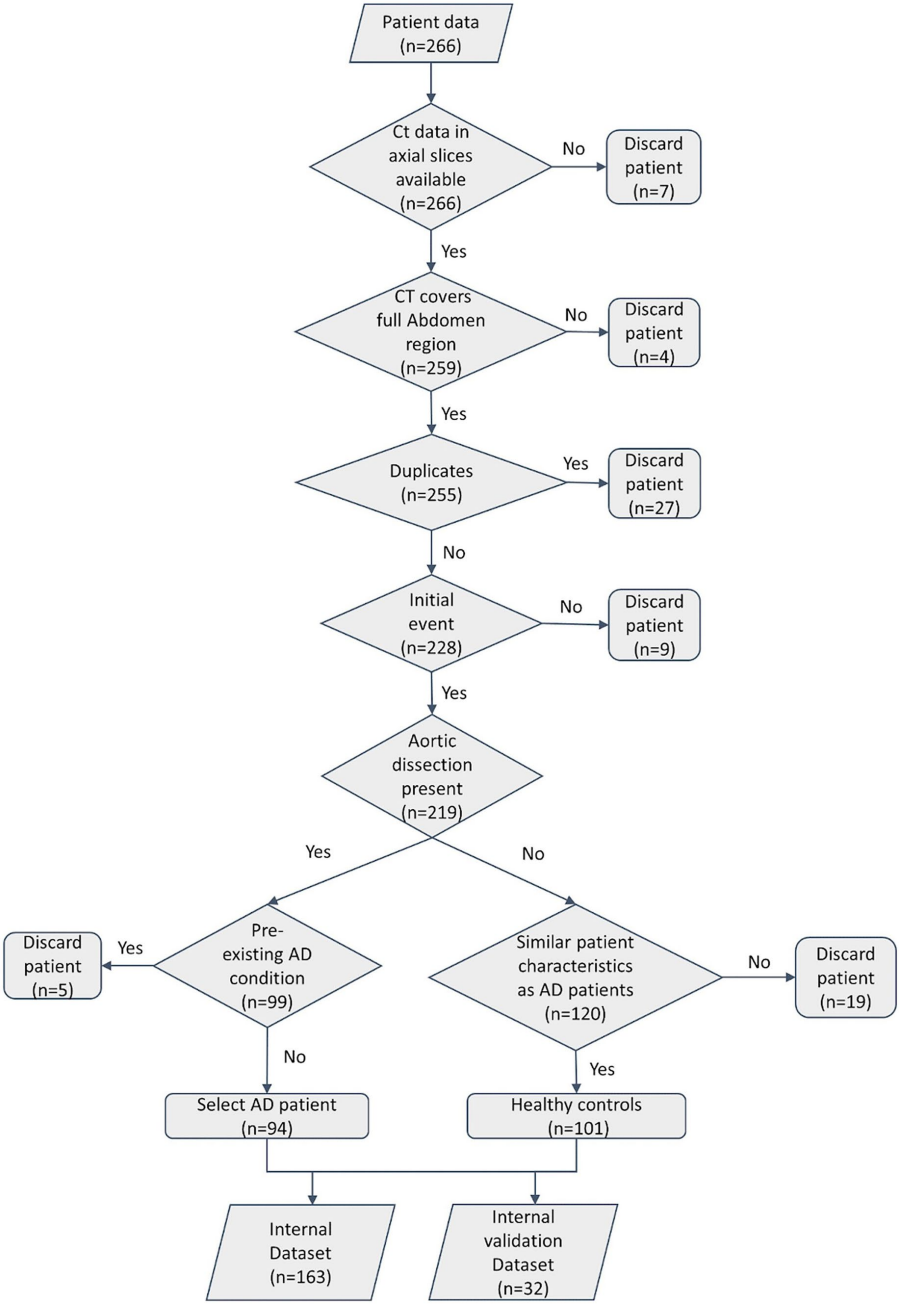
Internal dataset patient selection flowchart. Data acquisition process. The healthy controls (*n* = 101) were collected similarly to the included AD cases (*n* = 94)

From the total of *n* = 266 cases, *n* = 163 scans were used for training and *n* = 32 were used for validation, in the rest, either images were not available, or the abdominal aorta was not fully covered. AD patients on average were 65.9 ± 13.5 (29–93) years of age, 36% were female. For the training cases, in 72.0%, suprarenal AD was present, and infrarenal AD was seen in 79.3%. 9.8% presented with intramural hematoma, and 56.1% showed partial aortic lumen thrombosis. The external public dataset used for validation consisted of a dataset with *n* = 1189 cases (100 AD cases). Details are provided in Table 1 and patient selection criteria are shown in Fig. 4.

**Table 1:** Patient characteristics from internal and external set

	Internal set	Internal validation set	External validation set
Data source	Mannheim University Hospital, Germany		ImageTBAD, AVT dataset, Abdomen CT-1 k dataset (Details in the supplements)
Patient numbers	163 (78 AD)	32 (16 AD)	1189 (100 AD)
Patient age (years)	65.9 ± 13.5 (29–93)		AD: 52.20 ± 11.30 Non-AD: unknown
% Female	35,8%		AD: 40,7% Non-AD: unknown
Contrast phase	CTA 73.1%; venous: 26.9%		Mixed contrast
In-plane resolution (X/Y) (mm)	0.845 ± 0.106	0.845 ± 0.094	0.817 ± 0.120
Slice thickness (Z) (mm)	2.006 ± 1.341	1.836 ± 1.306	2.565 ± 1.538
Original image size (voxels)	512 × 512 × 495 ± 0 × 0 × 398	512 × 512 × 459 ± 0 × 0 × 228	512 × 514 × 224 ± 8 × 22 × 220

### Classification Results

From the four tested networks, our 5-layer CNN yielded the highest AUC (0.932) in cross-validation (Table 2) and was therefore chosen for further investigation. Its performance on internal and external validation sets are provided in Table 3. Furthermore, the confusion matrices and the AUC curves are illustrated in Fig. S1 (supplement) and 5, respectively. The entire workflow including preprocessing, aorta segmentation, and the prediction was tested on internal training (cross-validation) and validation dataset and takes approximately 45 s (model prediction = 0.003 s).

**Table 2:** Performance comparison of four different networks on the internal training dataset

Network	5-layer CNN (ours)	ResNet10	ResNet34 (pretrained)	SEResNet50
AUC	0.932	0.796	0.520	0.869

**Table 3:** Evaluation metrics for internal and external sets. The internal set results are from fivefold cross-validation test sets, while the external set results are from an ensemble of 5 models that were trained on the internal set. Furthermore, a separate internal validation set results from the ensemble of 5 models is shown

Dataset	AUC (95% CI)	Sensitivity	Specificity	Balanced accuracy (sensitivity + specificity/2)
Internal (cross-validation)	0.932 (0.891–0.963)	0.885 (69/78)	0.835 (71/85)	0.860 (1.720/2)
Internal (validation)	0.887 (0.732–0.988)	0.875 (14/16)	0.688 (11/16)	0.781 (1.563/2)
External (validation)	0.993 (0.988–0.997)	1.000 (100/100)	0.865 (942/1089)	0.933 (1.865/2)

#### Internal Set

**Fig. 5:**
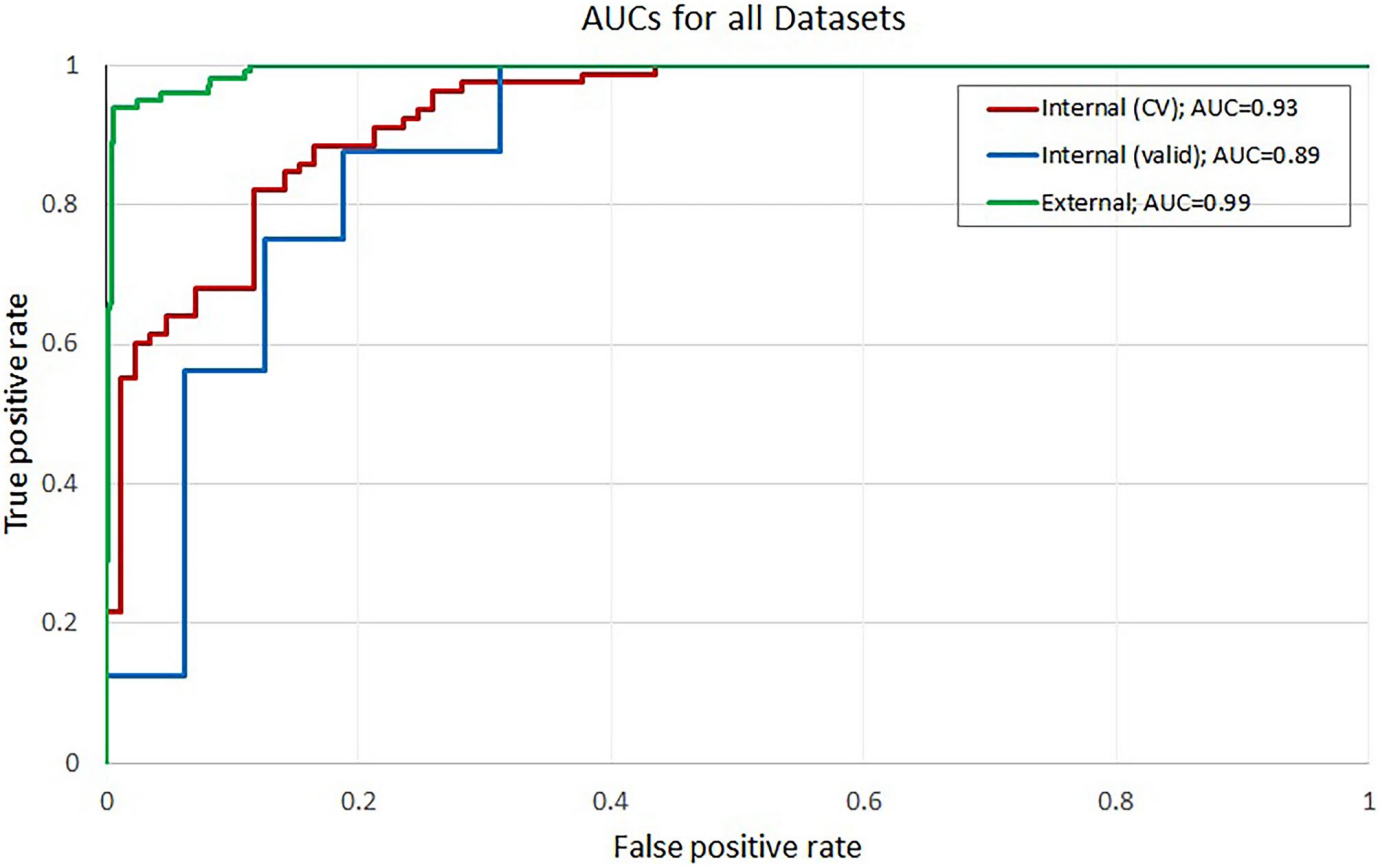
AUC curves for internal cross validation (CV), internal validation (valid), and external sets. The internal set AUC is 0.93, internal validation set AUC is 0.89, while the external set AUC is 0.99

The balanced accuracy score for the internal set fivefold cross-validation (test sets only) is 0.860 (Table 3). The corresponding sensitivity value is 0.885 (69/78), with the specificity being 0.835 (71/85) and the AUC of 0.932 (CI 0.891–0.963), which is shown in the confusion matrix (Fig. S1 (a)) and plotted in Fig. 5 (red). From the nine missed AD cases, six had a clearly visible AD and three presented with very subtle signs of AD (further described in supplement material and Fig. S4).

#### Internal Validation Set

The sensitivity and specificity for the internal validation set are 0.875 (14/16) and 0.688 (11/16), respectively, with a balanced accuracy of 0.789. The AUC value obtained is 0.887 (CI 0.732–0.988), as shown in Fig. S1 (b) and Fig. 5 (blue). Out of the five FP cases, two had a very subtle form of AD (examples in supplementary Fig. S3).

#### External Set

For the external validation dataset, a balanced accuracy score of 0.933 was obtained with sensitivity and specificity of 1.000 (100/100) and 0.865 (942/1089), respectively (Table 3, Fig. S1 (c)). Furthermore, the AUC value is 0.993 (CI 0.988–0.997) (Fig. 5 (green)).

## Discussion

This study demonstrated that rapid AI-based automated aortic dissection detection from CT images is feasible in an academic-level hospital in Germany. Overall, on all the datasets, an AUC of > 88.7% and sensitivities and specificities of > 87.5% and > 68.8% were consistently achieved. The strengths of this study are the comparably low training effort, and testing on a heterogeneous, real-world internal and external dataset with good overall processing times, indicating promising potential for clinical implementation as an alarming system for AD management.

AI-based approaches have been demonstrated to be of great potential for the detection of various pathologies in abdominal emergency imaging [9] and to support the management of chronic and acute vascular pathologies [27]. Structured text-based clinical data and imaging data have both been leveraged for AD detection, its rupture risk assessment, segmentation, therapy planning and prediction of mortality [28–30]. AI-based regular chest radiography analysis has been shown to offer a precision of 90.2% in the detection of AD [31]. Early approaches of CT imaging-based AD characterization tools used rule-based technologies and small datasets of *n* < 20 ADs [32]. Recently, CNN-based algorithms were proposed as mentioned in the introduction section. In contrast to most other algorithms, this study focussed on the abdominal region and comparably heterogeneous data with respect to CT scanners, contrast phase, and morphological characteristics of AD, resulting in sensitivity from 87.5 to 100% and specificity from 68.8 to 86.5%. It is important to mention that when used as a detection algorithm to improve prioritization of AD-positive cases, high sensitivity remains paramount and optimizing thresholds accordingly represents a very important design decision, but it also comes with the cost of a higher false-positive rate. An understanding of human performance is of high interest when interpreting AI performance. Nienaber et al. found the human sensitivity and specificity for the detection of acute AD in the thoracic region to be 93.8% and 87.1% [33], which is further underlined by the findings of Dreisbach et al., which describe substantial error rates of CT reading in acute AD under emergency conditions, depending on reader experience [6]. An important goal for further research is therefore to create a detailed understanding of the performance of human performance alone, but also with AI support, under realistic conditions in a prospective setup.

Clinical implementation of AI lagged behind expectations in the past [34]. The reasons are limited performance, lack of trust in AI systems, and poor workflow integration, amongst others [35]. A practical way of implementing this algorithm would be to automatically prioritize acute AD cases by re-ordering radiology reading lists [30] which has been shown to reduce delays in pulmonary embolism management [36] or to alarm specialized vascular care teams. Importantly, physicians would realistically expect the algorithm to not miss any cases of AD and a failure in this area can be expected to negatively impact trust in a clinical setting, therefore optimization of parameters contributing to sensitivity, but also the quality of user training require high attention. Moreover, a broader AI solution also covering chronic AD, PAU, and IMH would increase clinical use and therefore should be added with priority. To overcome the black box problem, a graphical visualization of the dissection membrane within the aorta in each detected case could offer substantial merit.

The results of this study are limited by various factors. First, due to the retrospective nature, there was an inevitable selection bias. As AD remains a rare condition, the dataset inevitably contains a limited number of patient cases. This limitation is particularly evident in our internal cases and extends to the variety of CT scanners and morphological differences between cases. Even though thorough external validation on a newly created dataset was performed, generalizability required confirmation and prospective multicenter testing with more patient cases therefore represents an important next goal. Additionally, the generation of synthetic training data using latent diffusion models could contribute to overcome these limitations.

Second, even though results are promising, balancing sensitivity and specificity remains a major issue. External validation demonstrated perfect sensitivity, but due to limited specificity, over half of the positive cases would yield false-positive results. On the internal training and validation datasets, on the other hand, six and two cases of AD were not detected. While some of these cases contained very subtle ADs and may have been missed for anatomical reasons, others presented with clear ADs where, for example, the isolation of the aortic vessel did not work correctly. Both the inclusion of larger training datasets as well as detailed refining and improvement of automated segmentation of the aortic lumen independent of external components like TotalSegmentator or Canny detector (for AD cases) might help improve performance. Third, due to the design-decision to not create a segmentation-based algorithm, possibilities of graphical visualization remain limited which could in the future possibly be added. Last, the focus only on abdominal imaging and only on AD limit clinical benefits to patients which present with abdominal AD, and therefore in the future, the inclusion of the full range of vascular pathologies and the thoracic region is needed.

## Conclusion

In conclusion, the proposed algorithm yielded sensitivity > 87.5% for the detection of AD within heterogeneous abdominal CT scans, which could be confirmed on an internal as well as external validation dataset. It therefore seems promising as a detection tool for AD in the abdomen which offers the potential for earlier detection of AD, especially in patients with unclear symptoms, leading to improved management. Further in-hospital testing is encouraged and necessary.

## Supplementary Information

Below is the link to the electronic supplementary material.

Supplementary file1 (PDF 848 KB)

## Data Availability

Data generated or analyzed during the study are available from the corresponding author by reasonable request.

## Abbreviations

AD: Aortic dissection
AUC: Area under the receiver operating characteristic curve
CT: Computed tomography
CNN: Convolutional neural network
HU: Hounsfield units
ROI: Region of interest
CI: Confidence interval
TP: True positive
TN: True negative
FP: False positive
FN: False negative
IMH: Intramural hematoma
PAU: Penetrating atherosclerotic ulcer
