# Supplementary material for: Streamlining Acute Abdominal Aortic Dissection Management—An AI-based CT Imaging Workflow
Source: J Imaging Inform Med. 2024 Jun 12;37(6):2729–39. doi: 10.1007/s10278-024-01164-0 (PMC11612133; doi:10.1007/s10278-024-01164-0)
Supplement: Supplementary file 1 — Supplementary file1 (PDF 848 KB) [file 10278_2024_1164_MOESM1_ESM.pdf]

Supplementary Materials

Index

Methods Supplement **1**
- Data augmentation 1
- Training 1
- Implementation 2

Dataset details 2
Clinical details of AD (internal) training cases 3
Result supplement **4**
Exemplary images of small and subtle cases **6**
Analysis of false negatives on internal training set 7
References **9**

Methods Supplement

Data augmentation

During training, we apply random rotation, zoom, and translation on the training set. Random rotation is performed with rotation along the Z-axis in the range [-12°, 12°]. Random translation has a range of [-15, 15] voxels for both the X- and Y-axis, while the range for the Z-axis is [-5, 5] voxels. Lastly, random zoom has a zoom range of [0.8, 1.2]. Each linear transformation is applied with a probability of 30% in each iteration.

Training

We tested three different networks: ResNet10 [1], ResNet34 (pretrained on MedicalNet [2]) and SEResNet50 [3] with varying network depths, of which all yielded comparably inferior results to our 5 layer CNN (Table 2).

We follow a 5-fold stratified cross-validation approach to make sure that each training, validation, and test set has the same proportion of dissection and healthy patients. The data split (internal dataset) is in a disjoint manner (patient level) with the number of samples in train (72%), validation (8%), and test set (20%) being 117, 13, and 32-33, respectively. We train the network (with random initialization) for 150 epochs with a batch size of 8. We use Adam optimizer with a learning rate of 1e-4. The objective function is a weighted binary cross-entropy, where the weight for the AD class is $(1 - number\ of\ AD\ cases / total\ number\ of\ cases)$. We further apply L2-regularization on the network weights, having a penalty term of 5e-5. Afterward, we select the model with the lowest validation loss in each fold for application on corresponding test sets.

All the tested networks followed the same training regime, except the pretrained ResNet34 model. Since this model was pretrained on medical data, we trained it on whole volume instead of only aorta ROI.

Implementation

The experiments are performed using Pytorch 1.13.1 with Python 3.9.15. We use the Monai (1.0.1) framework [4] to perform data augmentation and model development. Network training and inference were performed on the Nvidia RTX A6000 with a VRAM of 48 GB.

Dataset details

**Public validation dataset**		
	**AD cases**	**Non-AD cases**
Source	**ImageTBAD** dataset, Chinese Guangdong Provincial Peoples' Hospital,	**AVT dataset** [5], originating from the KiTS19 Grand Challenge (excluded in our study), Rider Lung CT dataset [6] and cases from Chinese Dongyang Hospital<br><br>**Abdomen CT-1K** dataset [7] originating from 6 sources: Bilic et al. [8] , Heller et al. [9] , Simpson et al. [10] , Roth et al. [11], Roth et al. [12], Clark et al. [13]
number of cases	n=100	n=1189
Average age	52.5 ± 11.3	unknown
% female	31%	unknown
CT hardware	Philipps (77%) and Siemens (23%)	various
slice spacing (mm)	0.75	0.5/5/5 mm<br>and<br>0.625/0.625/2.5 mm<br>and<br>2/3/3 mm
Voxel size	0.25x0.25x0.25 mm^3	

Table S1: Patient characteristics and technical details on external datasets.

Clinical details of AD (internal) training cases

**Total cases**	78
**Female**	35,4%
**Age**	66.6 ± 13.39 (29-92)
**% presence of suprarenal / infrarenal AD**	72,0 / 79,3%
**% presence of visceral ischemia**	15,9%
**% Obstruction celiac trunk / superior / mesenteric artery / left / right renal artery / inferior mesenteric artery / right side / left side limb ischemia**	4,9% / 1,2% / 4,9% / 9,8% / 17,1% / 2,4% / 1,2%
**% presence of aortic intramural haematoma**	9,8%
**% presence of aortic (partial) thrombosis**	56,1%

Table S2: Details of aortic dissection cases in the internal set.

Result supplement

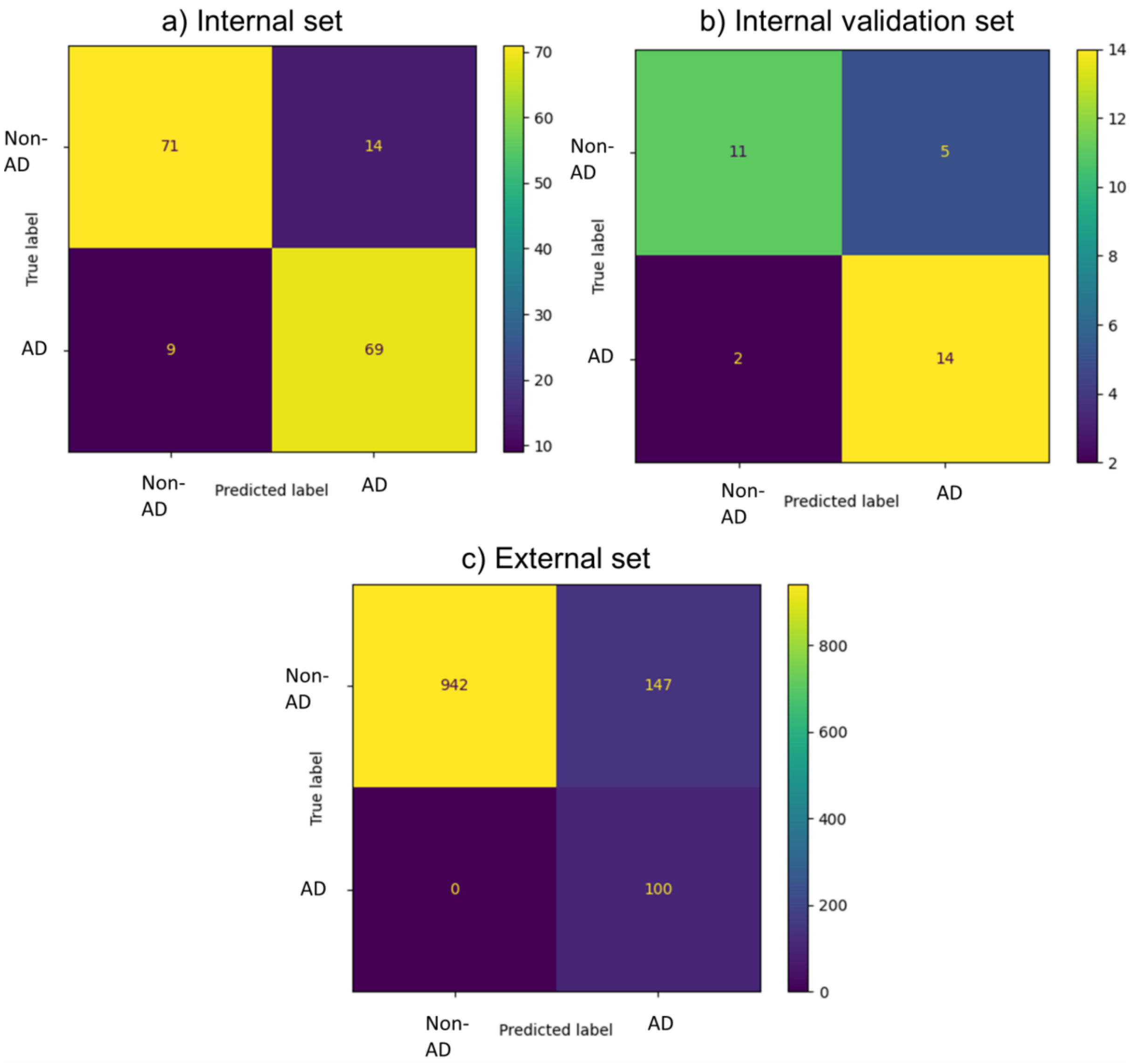

Figure S1: Confusion matrices for internal (cross validation), internal validation and external sets. A) Internal set cross-validation, B) internal validation set, and C) external set ensemble results.

Table S3 and Figure S2 depict ensemble performance on the external set for an optimal threshold value of 0.745. This threshold value was determined separately for the external dataset. Here, the sensitivity and specificity of 0.940 and 0.993 is achieved, respectively.

Dataset	Sensitivity	Specificity	Balanced accuracy (sensitivity + specificity / 2)	AUC (95% CI)
**External**	0.940 (94/100)	0.993 (1082/1089)	0.966 (1.993/2)	0.993 (0.988-0.997)

Table S3: External set evaluation metrics for an optimal threshold of 0.745.

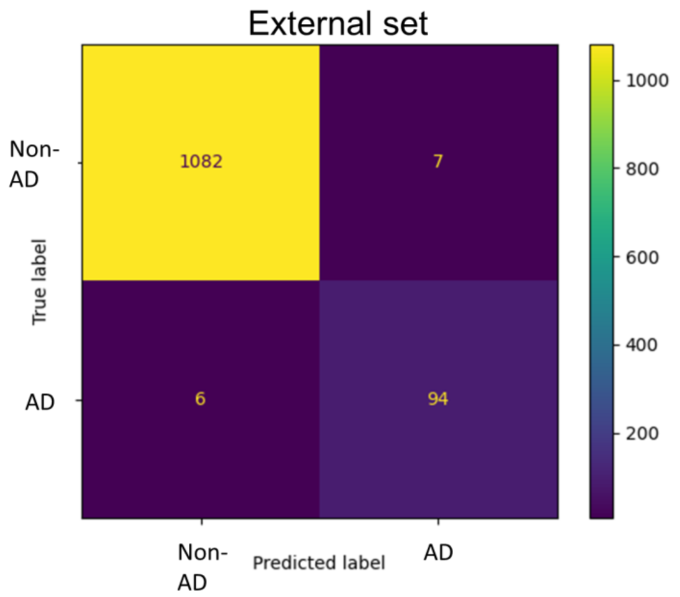

Figure S2: Confusion matrix for the external dataset with the optimal threshold of 0.745.

Exemplary images of small and subtle cases

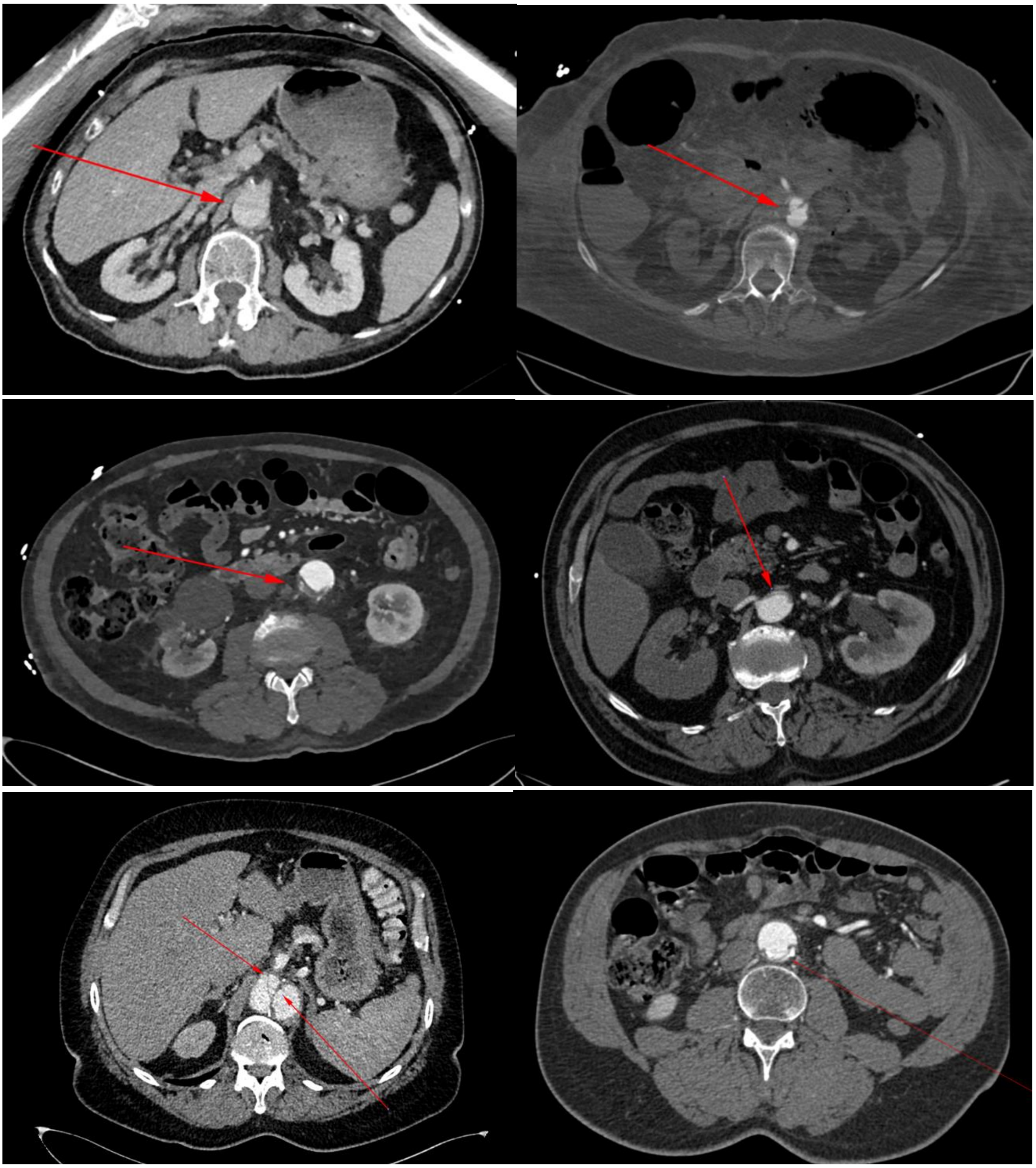

Figure S3. Array of atypical and subtle cases from the internal training and validation dataset.

Analysis of false negatives on internal training set

Wrong negatives and interpretation

Of the 9 FNs, 3 cases had unclear dissections. In these cases, the membrane was not clearly visible/subtle or there was no typical anatomy of dissection visible. In one case, only a small part of the abdomen was extracted, containing only a few slices of the dissected aorta. However, the other six cases had clear dissections. This could be due to the similarity in the aorta size of healthy and dissected cases in this case. The model might consider aorta of specific sizes to belong to a particular class, making it difficult for cases where the aorta sizes are similar in both the healthy and the dissected cases. The reasons behind this, however, remains unclear.

Figure S2 shows three example cases that were not classified as AD (FN). Interesting findings on each case in the order they appear are:

- Case 1: Dissection anatomy is not typical.
- Case 2: Only part of the abdomen was extracted, not covering a large part of the aorta. However, the dissection has a huge, very broad membrane.
- Case 3: The dissection is clear, and the aorta is large, yet the algorithm failed to correctly classify it.

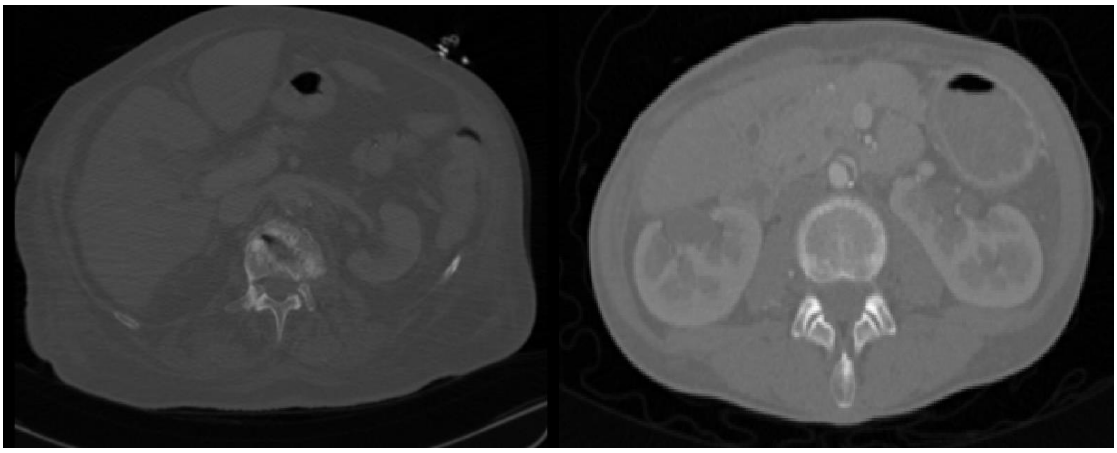

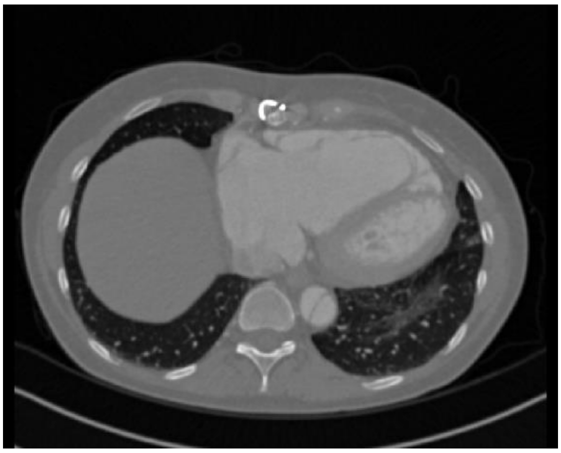

Figure S4: Example cases that were falsely classified as non-AD (FN).

References

1. Lee DK, Kim JH, Oh J, Kim TH, Yoon MS, Im DJ, Chung JH, Byun H (2022) Detection of acute thoracic aortic dissection based on plain chest radiography and a residual neural network (Resnet). Sci Rep 12:21884. https://doi.org/10.1038/s41598-022-26486-3
2. Chen S, Ma K, Zheng Y (2019) Med3D: Transfer Learning for 3D Medical Image Analysis
3. Hu J, Shen L, Albanie S, Sun G, Wu E (2019) Squeeze-and-Excitation Networks
4. Cardoso MJ, Li W, Brown R, Ma N, Kerfoot E, Wang Y, Murrey B, Myronenko A, Zhao C, Yang D, Nath V, He Y, Xu Z, Hatamizadeh A, Myronenko A, Zhu W, Liu Y, Zheng M, Tang Y, Yang I, Zephyr M, Hashemian B, Alle S, Darestani MZ, Budd C, Modat M, Vercauteren T, Wang G, Li Y, Hu Y, Fu Y, Gorman B, Johnson H, Genereaux B, Erdal BS, Gupta V, Diaz-Pinto A, Dourson A, Maier-Hein L, Jaeger PF, Baumgartner M, Kalpathy-Cramer J, Flores M, Kirby J, Cooper LAD, Roth HR, Xu D, Bericat D, Floca R, Zhou SK, Shuaib H, Farahani K, Maier-Hein KH, Aylward S, Dogra P, Ourselin S, Feng A (2022) MONAI: An open-source framework for deep learning in healthcare
5. Radl L, Jin Y, Pepe A, Li J, Gsaxner C, Zhao F-H, Egger J (2022) AVT: Multicenter aortic vessel tree CTA dataset collection with ground truth segmentation masks. Data Brief 40:107801. https://doi.org/10.1016/j.dib.2022.107801
6. Zhao B, Schwartz LH, Kris MG (2015) Data From RIDER Lung CT
7. Ma J, Zhang Y, Gu S, Zhu C, Ge C, Zhang Y, An X, Wang C, Wang Q, Liu X, Cao S, Zhang Q, Liu S, Wang Y, Li Y, He J, Yang X (2022) AbdomenCT-1K: Is Abdominal Organ Segmentation a Solved Problem? IEEE Trans Pattern Anal Mach Intell 44:6695–6714. https://doi.org/10.1109/TPAMI.2021.3100536
8. Bilic P, Christ P, Li HB, Vorontsov E, Ben-Cohen A, Kaissis G, Szeskin A, Jacobs C, Mamani GEH, Chartrand G, Lohöfer F, Holch JW, Sommer W, Hofmann F, Hostettler A, Lev-Cohain N, Drozdzal M, Amitai MM, Vivanti R, Sosna J, Ezhov I, Sekuboyina A, Navarro F, Kofler F, Paetzold JC, Shit S, Hu X, Lipková J, Rempfler M, Piraud M, Kirschke J, Wiestler B, Zhang Z, Hülsemeyer C, Beetz M, Ettlinger F, Antonelli M, Bae W, Bellver M, Bi L, Chen H, Chlebus G, Dam EB, Dou Q, Fu C-W, Georgescu B, Giró-i-Nieto X, Gruen F, Han X, Heng P-A, Hesser J, Moltz JH, Igel C, Isensee F, Jäger P, Jia F, Kaluva KC, Khened M, Kim I, Kim J-H, Kim S, Kohl S, Konopczynski T, Kori A, Krishnamurthi G, Li F, Li H, Li J, Li X, Lowengrub J, Ma J, Maier-Hein K, Maninis K-K, Meine H, Merhof D, Pai A, Perslev M, Petersen J, Pont-Tuset J, Qi J, Qi X, Rippel O, Roth K, Sarasua I, Schenk A, Shen Z, Torres J, Wachinger C, Wang C, Weninger L, Wu J, Xu D, Yang X, Yu SC-H, Yuan Y, Yue M, Zhang L, Cardoso J, Bakas S, Braren R, Heinemann V, Pal C, Tang A, Kadoury S, Soler L, van Ginneken B, Greenspan H, Joskowicz L, Menze B (2023) The Liver Tumor Segmentation Benchmark (LiTS). Med Image Anal 84:102680. https://doi.org/10.1016/j.media.2022.102680
9. Heller N, Isensee F, Maier-Hein KH, Hou X, Xie C, Li F, Nan Y, Mu G, Lin Z, Han M, Yao G, Gao Y, Zhang Y, Wang Y, Hou F, Yang J, Xiong G, Tian J, Zhong C, Ma J, Rickman J, Dean J, Stai B, Tejpaul R, Oestreich M, Blake P, Kaluzniak H, Raza S, Rosenberg J, Moore K, Walczak E, Rengel Z, Edgerton Z, Vasdev R, Peterson M, McSweeney S, Peterson S, Kalapara A, Sathianathen N, Papanikolopoulos N, Weight C (2021) The state of the art in kidney and kidney tumor segmentation in contrast-enhanced CT imaging: Results of the KiTS19 challenge. Med Image Anal 67:101821. https://doi.org/10.1016/j.media.2020.101821
10. Simpson AL, Antonelli M, Bakas S, Bilello M, Farahani K, van Ginneken B, Kopp-Schneider A, Landman BA, Litjens G, Menze B, Ronneberger O, Summers RM, Bilic P, Christ PF, Do RKG, Gollub M, Golia-Pernicka J, Heckers SH, Jarnagin WR, McHugo MK, Napel S, Vorontsov E, Maier-Hein L, Cardoso MJ (2019) A large annotated medical image dataset for the development and evaluation of segmentation algorithms
11. Roth H, Farag A, Turkbey EB, Lu L, Liu J, Summers RM (2016) Data From Pancreas-CT
12. Roth HR, Lu L, Farag A, Shin H-C, Liu J, Turkbey EB, Summers RM (2015) DeepOrgan: Multi-level Deep Convolutional Networks for Automated Pancreas Segmentation. In: Navab N, Hornegger J, Wells WM, Frangi A (eds) Medical Image Computing and Computer-Assisted Intervention -- MICCAI 2015. Springer International Publishing, Cham, pp 556–564
13. Clark K, Vendt B, Smith K, Freymann J, Kirby J, Koppel P, Moore S, Phillips S, Maffitt D, Pringle M, Tarbox L, Prior F (2013) The Cancer Imaging Archive (TCIA): Maintaining and Operating a Public Information Repository. J Digit Imaging 26:1045–1057.

https://doi.org/10.1007/s10278-013-9622-7
